# Inhibition of STAT5A promotes osteogenesis by DLX5 regulation

**DOI:** 10.1038/s41419-018-1184-7

**Published:** 2018-11-14

**Authors:** Kyoung-Mi Lee, Kwang Hwan Park, Ji Suk Hwang, Moses Lee, Dong Suk Yoon, Hyun Aae Ryu, Ho Sun Jung, Ki Won Park, Jihyun Kim, Sahng Wook Park, Sung-Hwan Kim, Yong-Min Chun, Woo Jin Choi, Jin Woo Lee

**Affiliations:** 1 Department of Orthopaedic Surgery, Yonsei University College of Medicine, 50-1 Yonsei -ro, Seodaemun-gu, Seoul, 03722 South Korea; 2 Severance Biomedical Science Institute, Yonsei University College of Medicine, 50-1 Yonsei -ro, Seodaemun-gu, Seoul, 03722 South Korea; 3 Department of Internal Medicine, Brody School of Medicine at East Carolina University, Greenville, NC 27834 USA; 4 Brain Korea 21 PLUS Project for Medical Sciences, Yonsei University College of Medicine, 50-1 Yonsei-ro, Seodaemun-gu, Seoul, 03722 South Korea; 5 Department of Biochemistry and Molecular Biology, Institute of Genetic Science, Integrated Genomic Research Center for Metabolic Regulation, Yonsei University College of Medicine, 50-1 Yonsei -ro, Seodaemun-gu, Seoul, 03722 South Korea

## Abstract

The regulation of osteogenesis is important for bone formation and fracture healing. Despite advances in understanding the molecular mechanisms of osteogenesis, crucial modulators in this process are not well-characterized. Here we demonstrate that suppression of signal transducer and activator of transcription 5A (STAT5A) activates distal-less homeobox 5 (DLX5) in human bone marrow-derived stromal cells (hBMSCs) and enhances osteogenesis in vitro and in vivo. We show that STAT5A negatively regulates expression of *Dlx5* in vitro and that STAT5A deletion results in increased trabecular and cortical bone mass and bone mineral density in mice. Additionally, STAT5A deletion prevents age-related bone loss. In a murine fracture model, STAT5A deletion was found to significantly enhance bone remodeling by stimulating the formation of a fracture callus. Our findings indicate that STAT5A inhibition enhances bone formation by promoting osteogenesis of BMSCs.

## Introduction

Human bone marrow mesenchymal stromal cells (hBMSCs) may differentiate into osteoblasts, chondrocytes, adipocytes, or tenocytes depending on culture conditions. Among these, osteoblast differentiation is important for increasing bone mass. Osteoblast differentiation of hBMSCs is a well-characterized process and proceeds as a result of the timely expression of genes, such as alkaline phosphatase (*ALP*), runt-related transcription factor 2 (*RUNX2*), distal-less homeobox 5 (*DLX5*), osterix (*OSX*), and osteocalcin (*OCN*), followed by extracellular matrix synthesis and mineralization^1,2^. RUNX2 has been characterized as the master transcription factor in osteogenic differentiation and bone formation^3,4^. Another transcription factor, DLX5, is also expressed in osteoblasts. DLX5 is an important regulator in the development of mineralized tissues because it induces expression of RUNX2 in the bone morphogenetic protein (BMP) signaling pathway^5^. DLX5 is also involved in osteoblast maturation^6^, and *Dlx5*^-/-^ mice have been found to have severe craniofacial abnormalities with delayed ossification of the dermatocranial bones^7^. These findings suggest that the *Dlx5* gene may play a role in bone formation and fracture healing.

Members of the signal transducers and activators of transcription (STAT) family play important roles in cell proliferation, differentiation, and survival via effects on the expression of cytokines, growth factors, and hormones^8–11^. Like other STAT family members, STAT5 was originally identified as a cytosolic signaling molecule involved in proliferation, differentiation, and apoptosis in cancer cell lines^12,13^. Following stimulation by various cytokines, STAT5 becomes phosphorylated and forms a dimer^14,15^. The STAT5 dimer then translocates into the nucleus where it may bind to interferon-γ-activated sequence (GAS) motifs, promoting the transcription of target genes^9,16,17^. There are two isoforms of STAT5: STAT5A and STAT5B^18^. Interestingly, STAT5A and STAT5B are encoded by separate genes, but the proteins are 90% identical at the amino acid level^14^. The distinct functions of these isoforms have been explored in several in vivo studies by using STAT5 global knockout mice^19^. The prolactin-mediated functions are blocked in the mammary gland by STAT5A^-/-^^10,20^, while STAT5B^-/-^ disrupts the functions that are regulated by the growth hormone in the liver^11,21^. Mostly, the studies about STAT5 global knockout mice were conducted in hematopoietic stem cells^20,22,23^.

Recently, STAT5 was found to function as a negative regulator of bone resorption in osteoclasts in vitro and in vivo^24^. Another study reported that STAT5–RUNX2 interaction promotes osteoblast differentiation in vitro^25^. However, no study has yet distinguished the roles in osteoblast differentiation played by the different isoforms of STAT5. Previously, Jung et al. demonstrated that STAT5A plays a major role in adipogenesis of hBMSCs and STAT5B performs only a supportive function in these processes^26^. These results suggest that STAT5A plays an essential role in maintaining bone homeostasis by balancing osteogenesis and adipogenesis. In this study, we aimed to characterize the role of STAT5A in osteoblasts. By clarifying which STAT5 isoform contributes to osteoblast differentiation, our findings might identify a novel therapeutic target for bone diseases.

In this study, we found that STAT5A played an important role in bone formation and regeneration. We found that inhibition of STAT5A promoted osteoblast differentiation and bone formation through the activation of DLX5 signaling. STAT5A deletion was found to result in significantly decreased bone loss in a murine age-related osteoporosis model. Moreover, in a murine fracture model, we found that STAT5A deletion enhanced bone healing by stimulating new bone formation. These findings support an inhibitory function for STAT5A in osteogenesis and suggest therapeutic potential for STAT5A inhibition in age-related osteoporosis and fracture healing.

## Results

### Overexpression of STAT5A suppresses osteogenesis in hBMSCs

**Fig. 1:**
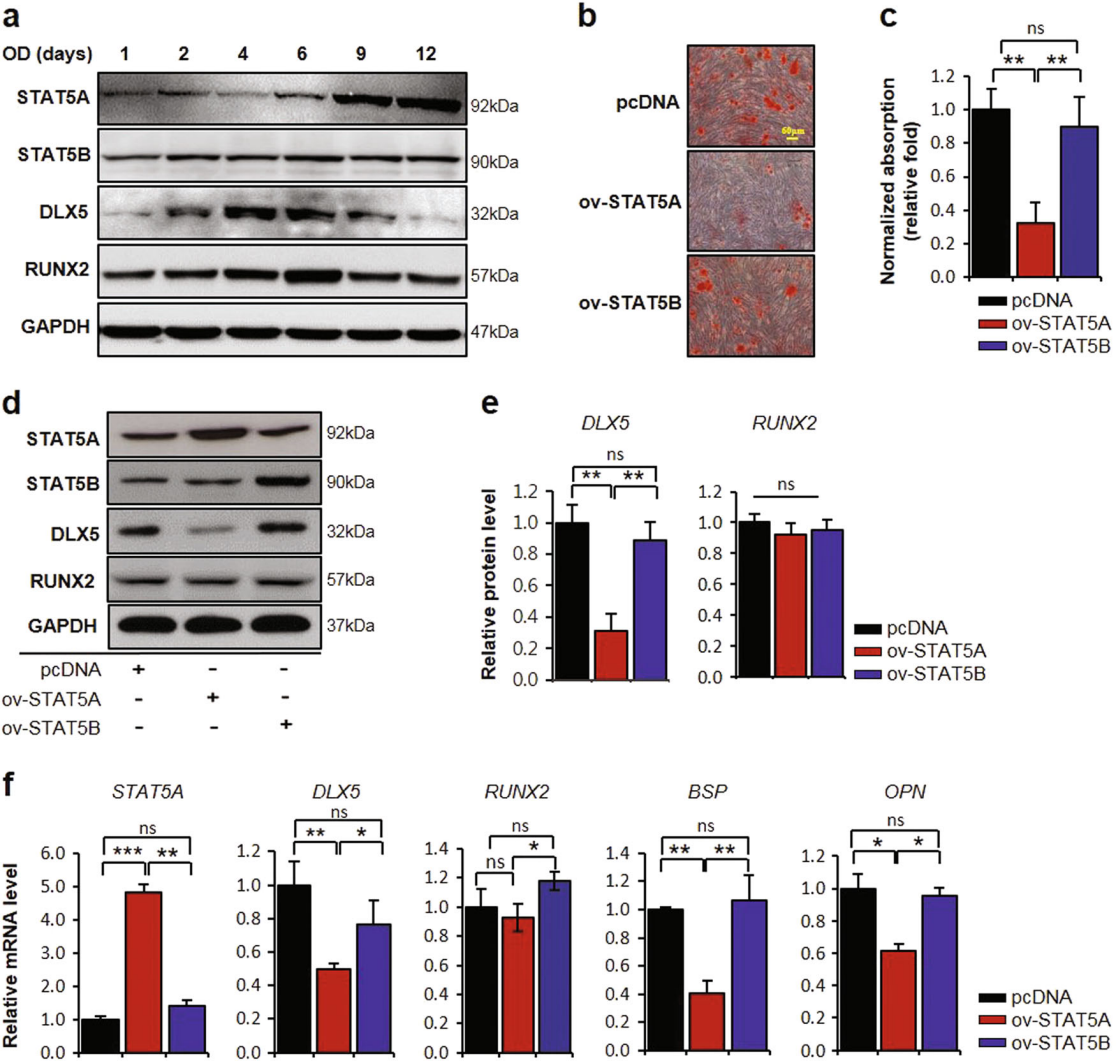
Suppressed osteoblastic differentiation of hBMSCs by overexpression of STAT5A. **a** Protein levels of STAT5A, STAT5B, DLX5, and RUNX2 during osteogenesis of hBMSCs. **b** Alizarin Red S staining demonstrating the effect of STAT5 overexpression on the osteogenesis at day 14. Scale bar, 60 μm. **c** Quantification of Alizarin Red S staining. **d** Western blot analysis of protein expression of osteogenic master genes, DLX5 and Runx2 after STAT5 overexpression. GAPDH was used as a control. **e** Quantification of DLX5 and RUNX2 protein levels. **f** Gene expression of DLX5 and expression of downstream genes of DLX5 after 4 days with STAT5 overexpression in hBMSCs using real-time PCR. All mRNA and protein levels were normalized with GAPDH. Each experiment was performed in triplicate (*n* = 3). All error bars indicate ± SEM. *P < 0.01; **P < 0.01; ***P < 0.001

To investigate the possible role of STAT5 during osteogenesis, we first examined STAT5 isoform expression during osteogenesis in hBMSCs. Interestingly, STAT5A was found to increase in response to induction of osteogenesis after 6 days (Fig. 1a). However, there was no change in the expression of STAT5B during osteogenesis, while DLX5 and RUNX2, which are key osteogenic markers, were decreased in the later stage of osteogenesis. To examine the effects of STAT5A and STAT5B on osteogenesis, we overexpressed STAT5A and STAT5B in hBMSCs. On day 14 after the induction of osteogenesis, mineralization of STAT5A-overexpressed hBMSCs was significantly decreased compared to that of control hBMSCs and STAT5B-overexpressed hBMSCs (Fig. 1b, c). Following Alizarin Red S staining and quantification, we also confirmed protein levels for the osteogenic marker genes DLX5 and RUNX2. Overexpression of STAT5A in hBMSCs was found to result in significantly decreased DLX5 expression but not decreased RUNX2 expression. Overexpression of STAT5B, however, had no effect on these proteins (Fig. 1d, e). Accordingly, STAT5A decreased messenger RNA (mRNA) levels of *DLX5* and its downstream genes coding for bone sialoprotein (*BSP*) and osteopontin (*OPN*) but the mRNA of *RUNX2* was not changed (Fig. 1f). These findings suggest that STAT5A functions during osteogenesis of hBMSCs via regulation of DLX5 expression.

### Suppression of STAT5A promotes osteogenesis in hBMSCs

**Fig. 2:**
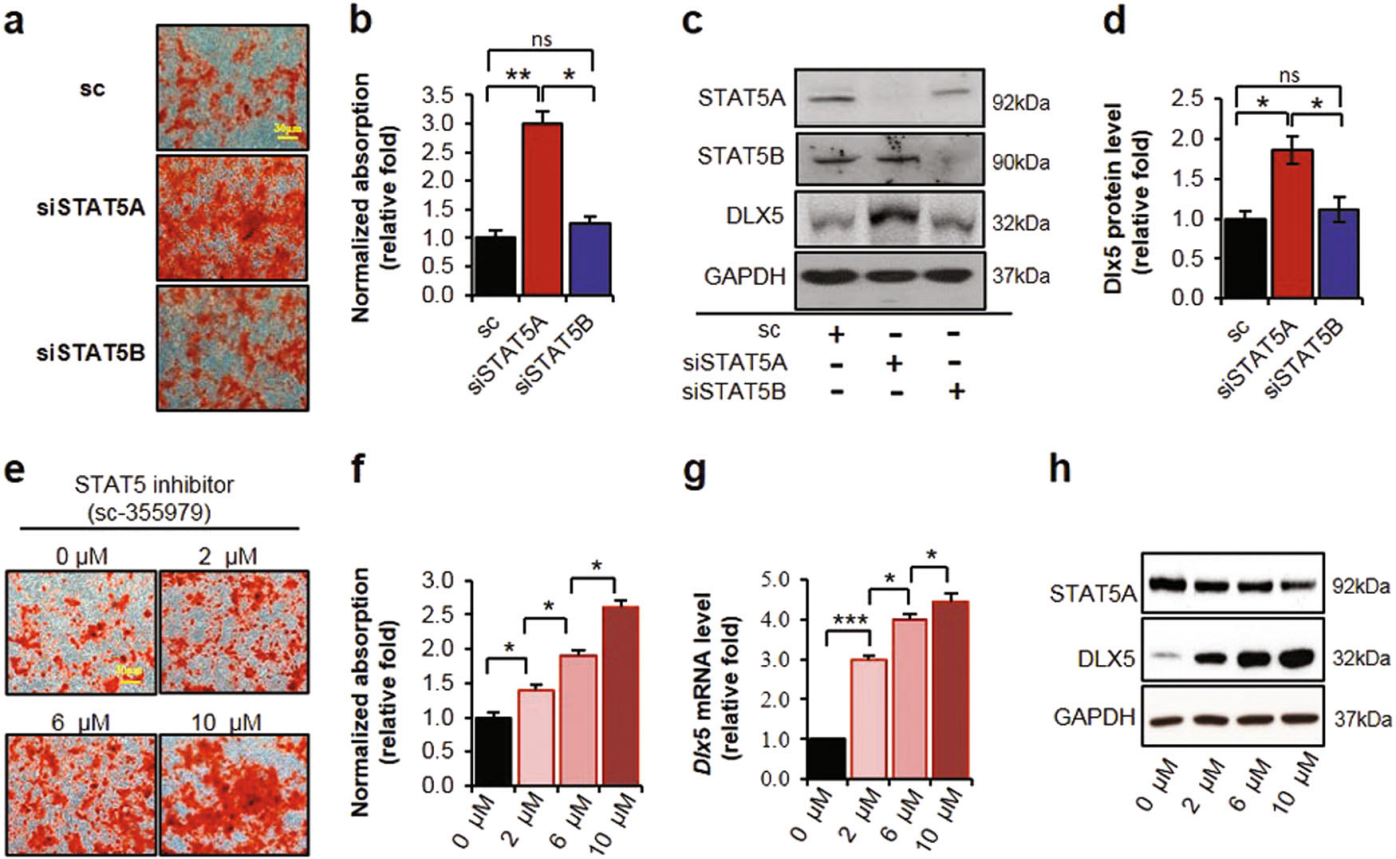
Induced osteoblastic differentiation through increased DLX5 expression by inhibition of STAT5A in hBMSCs. **a** Alizarin Red S staining to detect suppression of STAT5A and STAT5B on osteogenesis using targeted siRNA. Staining was performed on day 14 of osteogenesis. Scale bar, 30 μm. **b** Quantification of Alizarin Red S staining. **c** Western blot analysis of DLX5 protein expression after silencing of STAT5A in hBMSCs. **d** Quantification of DLX5 protein level compared with GAPDH expression level. **e** Alizarin Red S staining for the effect of STAT5 inhibitor during osteogenesis of hBMSCs. STAT5 inhibitor (sc-355797) was used at concentrations of 0, 2, 6, and 10 μM. Staining was performed at day 14 of osteogenesis. Scale bar, 30 μm. **f** Quantification of Alizarin Red S staining. **g** Analysis of DLX5 mRNA level after 10 μM STAT5 inhibitor treatment. mRNA level was checked on day 5 of osteogenesis. **h** Western blot analysis of DLX5 protein expression after STAT5 inhibitor treatment. Protein level was detected on day 5 of osteogenesis. Each experiment was performed in triplicate (*n* = 3). All error bars indicate ±SEM. *P < 0.01; **P < 0.01; ***P < 0.001

To clarify the distinct roles of STAT5A and STAT5B in osteogenesis of hBMSCs, we knocked down STAT5A and STAT5B using small interfering RNAs (siRNAs), and we also treated hBMSCs with a STAT5 inhibitor (sc-355979). Suppression of STAT5A by siRNA had no effect on mRNA and protein expression of STAT5B (Supplementary Figures 1a and b). The siRNA-mediated knockdown of STAT5A promoted osteoblast differentiation, while siRNA-mediated knockdown of STAT5B had no effect (Fig. 2a, b). We then tested whether STAT5A regulates the expression of DLX5. The siRNA-mediated knockdown of STAT5A increased DLX5 protein expression (Fig. 2c, d). Consistently, a STAT5 inhibitor-induced mineral accumulation in a dose-dependent manner at day 14 after the induction of osteogenesis (Fig. 2e). At the dose for which the STAT5 inhibitor exhibited its maximal effect (10 μM), mineral accumulation was increased about 2.5-fold over that in vehicle control-treated cells (Fig. 2f). Accordingly, the STAT5 inhibitor increased *DLX5* mRNA and protein expression in a dose-dependent manner in hBMSCs (Fig. 2g, h).

### STAT5A directly regulates DLX5 in hBMSCs

To examine the relationship between STAT5A and the osteogenic transcriptional regulator DLX5 luciferase assays for each regulator were performed at day 5 after induction of osteogenesis in hBMSCs. STAT5A reduced *DLX5* promoter activity in a dose-dependent manner (Supplementary Figure 2a). Furthermore, *DLX5* promoter assays showed that the STAT5 inhibitor partially restored *DLX5* promoter activity that had been decreased by STAT5A (Supplementary Figure 3b).

To characterize the mechanism by which STAT5A suppresses *DLX5* promoter activity, we first predicted a STAT5A-binding site within the *DLX5* promoter region (Supplementary Figure 3c, asterisk) using the transcription element search system (TESS). We then designed truncated constructs of the *DLX5* promoter (Supplementary Figure 3c). We observed significantly decreased *DLX5* promoter activity in constructs containing the STAT5A-binding site relative to vector control, vs. no change was seen in the activities of constructs without a STAT5A-binding site (Supplementary Figure 3d). Furthermore, chromatin immuno-precipitation (ChIP) assays demonstrated that osteogenic stimulation for 5 days induced recruitment of STAT5A to the *DLX5* promoter, supporting the occurrence of direct regulation of DLX5 expression by STAT5A (Supplementary Figure 3e).

### Deletion of STAT5A enhances bone formation in mice

**Fig. 3:**
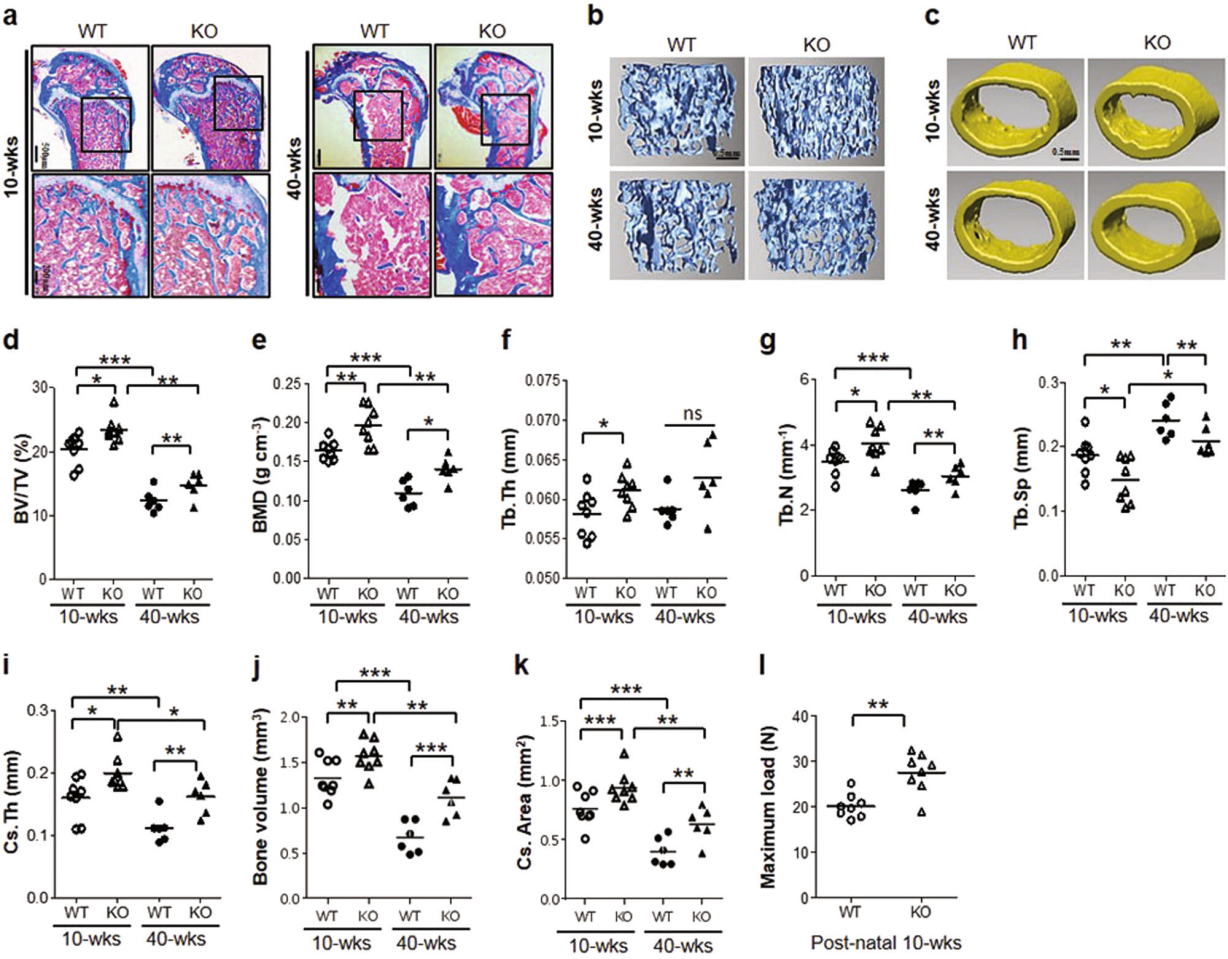
Increased bone formation of *Stat5a*^*-/-*^ mice. **a** Masson’s Trichrome staining of longitudinal sections of 10-week-old and 40-week-old male wild-type (WT) and *Stat5a*^*-/-*^ (KO) femurs. Scale bar: 500 μm (top) and 200 μm (bottom) (10-weeks: *n* = 8 and 40-weeks: *n* = 6 per each group). **b** Representative µCT images of trabecular bone from 10-week-old and 40-week-old male wild-type and *Stat5a*^*-/-*^ femurs. Scale bars, 0.5 mm. **c** Representative µCT images of cortical bone at 10 and 40 weeks of age in male wild-type and *Stat5a*^*-/-*^ femurs. Scale bars, 0.5 mm. **d**–**h** Quantitative µCT analysis of trabecular bone parameters for 10-week-old and 40-week-old male wild-type (WT) and *Stat5a*^*-/-*^ (KO) femurs, including (**d**) bone volume fraction ratio (BV/TV), (**e**) volumetric BMD of trabecular bone, (**f**) trabecular thickness (Tb.Th), (**g**) trabecular number (Tb.N), and (**h**) trabecular separation (Tb.Sp) are shown. (**i**–**k**) Quantitative µCT analysis of cortical bone parameters for 10-week-old and 40-week-old male wild-type (WT) and *Stat5a*^*-/-*^ (KO) femurs, with (**i**) cross-sectional thickness (Cs.Th), (**j**) bone volume, and (**k**) cross-sectional area (Cs.Area) are shown. **l** Maximum load on femurs from postnatal 10-week-old mice (*n* = 8 for each group). All error bars indicate ±SEM. *P <0.01; **P < 0.01; ***P < 0.001

To determine the in vivo role of STAT5A in skeletal development, we bred mice up to the seventh generation. Using whole-mount Alizarin Red/Alcian Blue-stained skeletal preparations, general aspects of skeletal development were not found to differ between E19.5 embryos of wild-type and *Stat5a*^*-/-*^ mice (Supplementary Figure S2). However, postnatal histologic analysis using Masson’s Trichrome staining revealed that femur trabecular bone mass was markedly increased in *Stat5a*^*-/-*^ mice compared to that in wild-type mice (Fig. 3a). Microcomputed tomography (μCT) analysis of femurs also showed that *Stat5a* deletion resulted in increased trabecular and cortical bone mass relative to wild-type mice (Fig. 3b, c). We found that multiple μCT parameters for trabecular bone were increased in *Stat5a*^*-/-*^ mice compared to wild-type mice (Fig. 3d–h). Also, we analyzed the difference between 10 and 40 weeks in the two genotypes. In *Stat5a*^*-/-*^ mice, the bone mineral density (BMD) reduction rate between 10 and 40 weeks was lower than that of wild-type mice. We found that *Stat5a* deletion reversed age-related reductions in bone mass and BMD in 40-week-old mice, suggesting that *Stat5a* deletion had a protective effect against age-related osteoporosis. Additionally, cortical thickness, one of the most important factors in determining mechanical bone strength, was significantly higher in *Stat5a*^*-/-*^ mice compared to wild-type mice (Fig. 3i). Cortical bone volume and cortical cross-sectional area were also higher in *Stat5a*^*-/-*^ mice compared to wild-type mice (Fig. 3j, k). Femoral shaft biomechanical properties were assessed by three-point bending, and *Stat5a* deletion resulted in significantly increased bone strength during bending compared to wild-type mice (Fig. 3l). Taken together, our results suggest a role for STAT5A in the maintenance of bone mass.

### STAT5A deletion promotes osteogenesis in mBMSCs

**Fig. 4:**
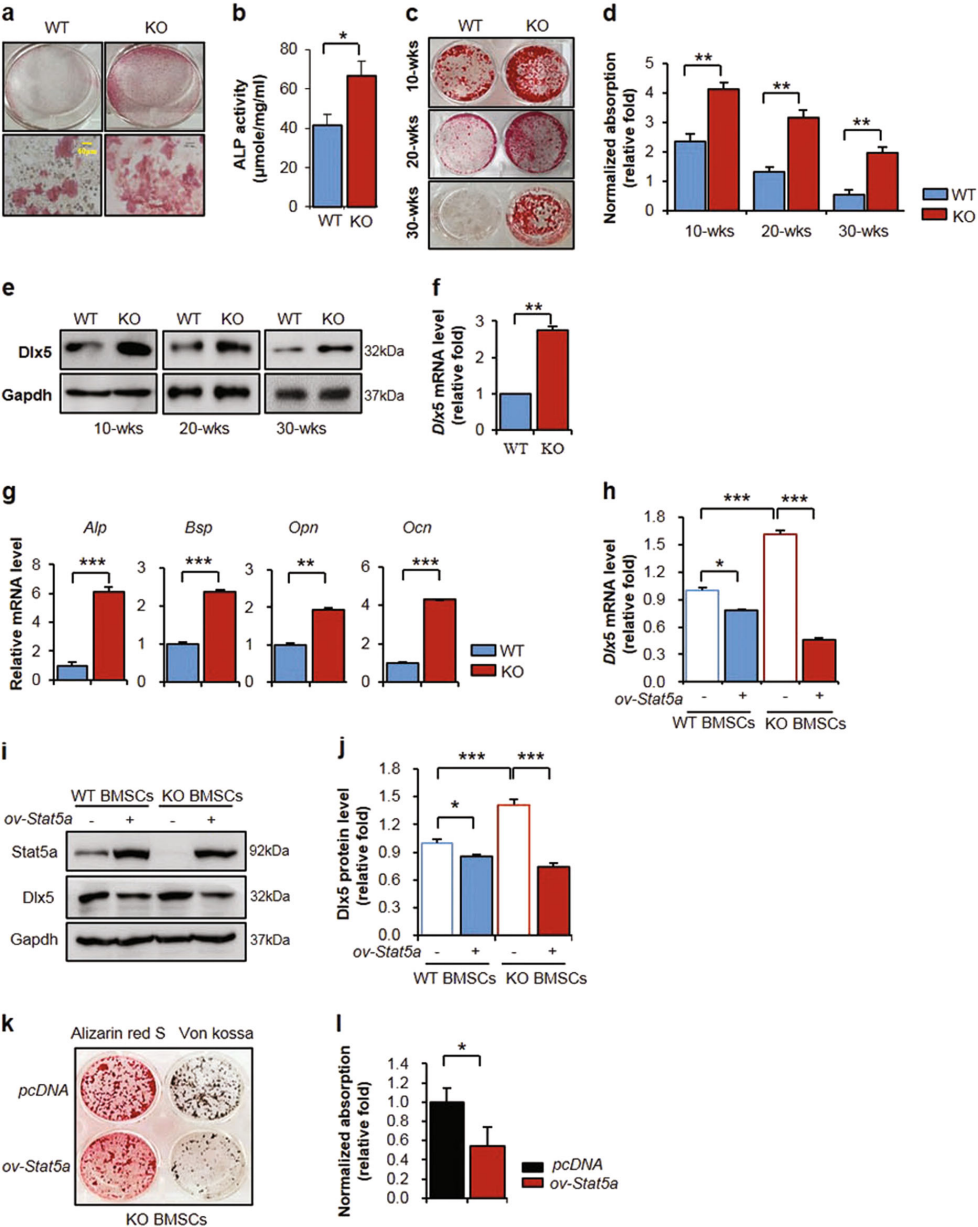
Induced osteoblast differentiation in *Stat5a*^*-/-*^ mBMSCs via upregulation of DLX5. **a** ALP staining of 10-week-old wild-type and *Stat5a*^*-/-*^ mBMSCs at day 0, as indicated. Scale bar, 60 μm. **b** Relative ALP activity assay results at days 0 in wild-type and *Stat5a*^*-/-*^ mBMSCs. **c** and **d** Alizarin Red S staining (**c**) and quantitative analysis (**d**) of at day 8 after induction of osteogenesis in 10-week-old, 20-week-old, and 30-week-old wild-type and *Stat5a*^*-/-*^ mBMSCs. **e** Protein levels of DLX5 in 10-week-old, 20-week-old, and 30-week-old male wild-type and *Stat5a*^*-/-*^ mBMSCs using western blotting at 5 days after osteogenesis. **f** mRNA levels of *Dlx5* in wild-type and *Stat5a*^*-/-*^ mBMSCs at day 3 after the induction of osteogenesis. **g** mRNA levels of osteoblast-related genes *Alp*, *Bsp, and Opn* at day 3 and mRNA level of *Ocn* at day 7 after the induction of osteogenesis. **h**–**j** Relative mRNA (**h**) and protein levels (**I** and **j**) of DLX5 depending on exogenously increased *Stat5a* expression in wild-type and *Stat5a*^*-/-*^ mBMSCs. pcDNA-mStat5a was transfected in *Stat5a*^*-/-*^ mBMSCs. mRNA and protein levels of *Dlx5* were checked at day 3 and 5 after osteogenesis, respectively. Osteogenesis was induced after overexpression of *Stat5a* by transfection. **k** The osteoblast differentiation of *Stat5a*^*-/-*^ mBMSCs upon overexpression of STAT5A using Alizarin Red S and Von Kossa staining at day 8 after induction of osteogenesis. **l** Quantification of Alizarin Red S staining in *Stat5a*^*-/-*^ mBMSCs. Each experiment was performed in triplicate (*n* = 3). All error bars indicate ±SEM. *P < 0.01; **P < 0.01; ***P < 0.001

To investigate the osteoblast activity of mouse bone marrow-derived stromal cells (mBMSCs), we isolated mBMSCs from wild-type and *Stat5a*^*-/-*^ mice. *Stat5a*^*-/-*^ mBMSCs showed significantly higher baseline ALP activity compared to wild-type mice (Fig. 4a, b). Regardless of age, mineral accumulations in *Stat5a*^*-/-*^ mBMSCs were markedly higher compared to wild-type mice at day 8 after the induction of osteogenesis (Fig. 4c). Interestingly, mineral accumulations in 30-week-old *Stat5a*^*-/-*^ mBMSCs were decreased compared to 10-week-old *Stat5a*^*-/-*^ mBMSCs, but comparable to those of 10-week-old wild-type mice (Fig. 4d). Accordingly, DLX5 protein and mRNA expression levels were higher in *Stat5*^*-/-*^ mBMSCs compared to those in wild-type mice (Fig. 4e, f). *Stat5a* deletion led to significant increases in mRNA expression of the osteogenic genes *Alp, Bsp, Opn*, and *Ocn* (Fig. 4g). Next, we examined whether STAT5A overexpression reversed DLX5 expression in *Stat5a*^*-/-*^ mBMSCs. STAT5A overexpression in *Stat5a*^*-/-*^ mBMSCs significantly decreased *Dlx5* mRNA and protein expression, which had been increased by STAT5A deletion (Fig. 4h–j). In accordance with these findings, *Stat5a* overexpression decreased mineral accumulation and calcification in *Stat5a*^*-/-*^ mBMSCs (Fig. 4k, l). Conversely, DLX5 suppression in *Stat5a*^*-/-*^ mBMSCs resulted in decreased osteogenesis (Supplementary Figure 4). These results demonstrate that STAT5A control osteogenic differentiation by regulating of DLX5.

### STAT5A deletion promotes bone fracture healing

**Fig. 5:**
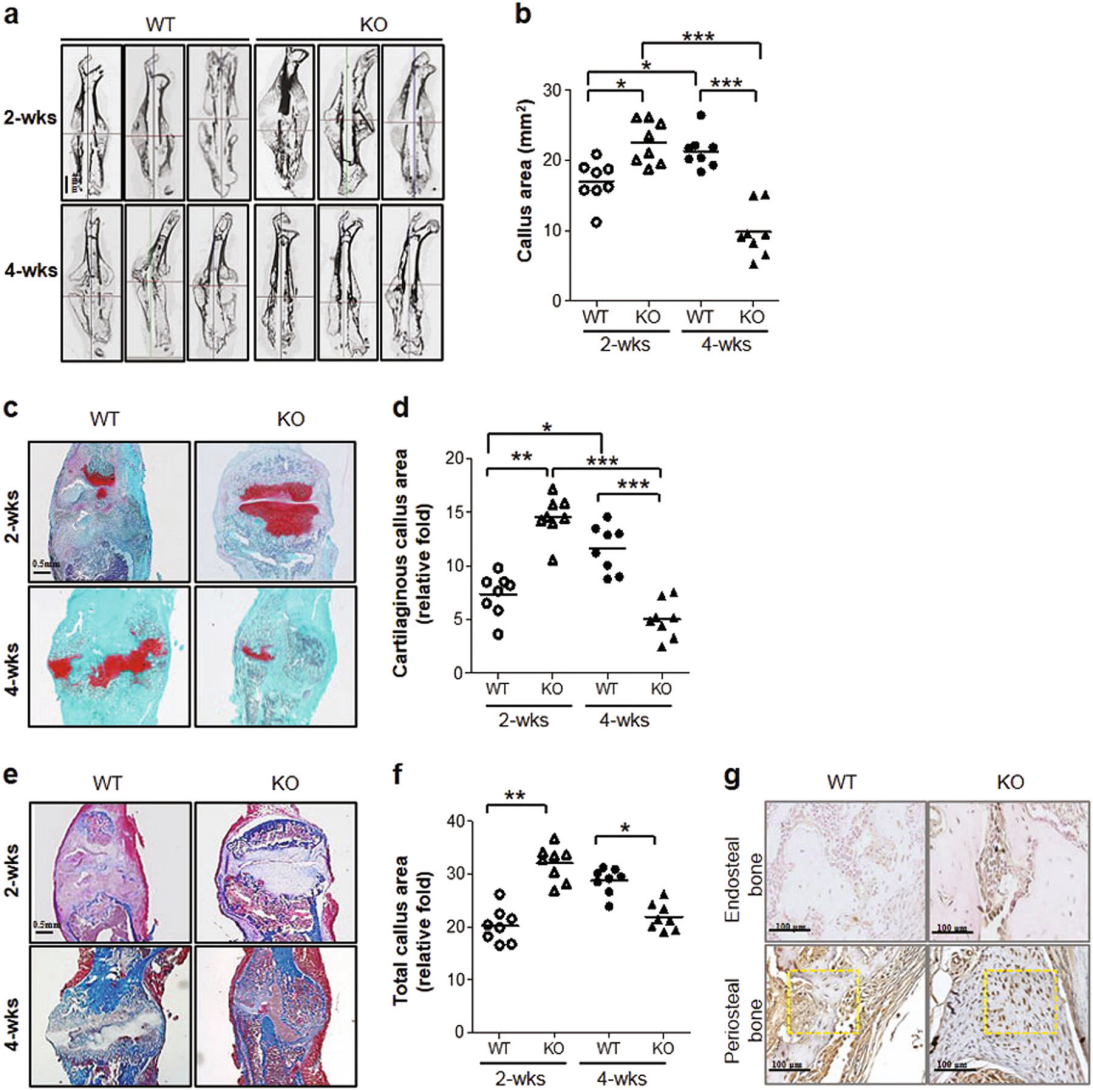
Enhanced bone fracture healing in *Stat5a*^*-/-*^ mice. **a** Representative longitudinal sections of fractured femurs from wild-type and *Stat5a*^*-/-*^ mice at 2 and 4 weeks post-fracture. 6-week-old male mice were used for the fracture model. Scale bar, 4 mm (*n* = 16 per each group). **b** Quantitative analysis of newly formed callus volume at 2 and 4 weeks post-fracture. **c**–**f** Representative histological analysis of paraffin sections of calluses from wild-type and *Stat5a*^*-/-*^ mice at 2 and 4 weeks post-fracture stained with (**c**) Safranin O/Fast Green staining for cartilaginous bone callus. **d** Quantitative analysis of remaining cartilaginous callus area at 2 and 4 weeks post-fracture (*n* = 8 per each group). Scale bar, 0.5 mm. **e** Masson’s Trichrome staining for total callus. **f** Quantitative analysis of total callus area at 2 and 4 weeks post-fracture (*n* = 8 per each group). Scale bar, 0.5 mm. **g** Immunohistochemistry against DLX5 at 2 weeks post-fracture of wild-type and *Stat5a*^*-/-*^ mice in the fractured femoral section. Scale bars, 100 μm (*n* = 5 for each group). All error bars indicate ±SEM. *P <0.01; **P <0.01; ***P <0.001

We next investigated the possible roles of STAT5A in fracture healing using a murine fracture model. In this experiment, we generated femur fractures in 6-week-old wild-type and *Stat5a*^*-/-*^ mice as previously described^27^. At 2 and 4 weeks post-procedure, we then examined callus formation using μCT (Fig. 5a). Analysis of μCT images found that callus area in *Stat5a*^*-/-*^ mice was increased at 2 weeks post-fracture and decreased at 4 weeks post-fracture, as compared to wild-type mice (Fig. 5b). Interestingly, the reduction rate of callus bone and cartilaginous callus in *Stat5a*^*-/-*^ mice were significantly higher than wild-type mice (Fig. 5b, c). Accordingly, cartilaginous callus stained with Safranin O and total callus stained with Masson’s Trichrome showed similar patterns to those found by μCT, suggesting that *Stat5a* deletion accelerated the fracture healing process in our model (Fig. 5c–f). Furthermore, immunohistochemical staining demonstrated that DLX5 expression was increased at the fracture site of *Stat5a*^*-/-*^ mice. Notably, DLX5 was highly expressed in periosteal bone at the fracture site in *Stat5a*^*-/-*^ mice (Fig. 5g). Overall, these results indicate that *Stat5a* deletion promotes bone regeneration and healing under fracture conditions.

### STAT5A deletion increased osteoclastogenesis in vivo

Next, we examined the effect of STAT5A on osteoclast differentiation in mouse bone marrow monocytes (mBMMs). mBMMs from wild-type and *Stat5a*^*-/-*^ mice were cultured for 3–4 days in *α*-minimum essential medium (α-MEM) with receptor activator of nuclear factor-kappa B ligand (RANKL) and macrophage colony stimulating factor (M-CSF). On day 4 after the induction of osteoclastogenesis, osteoclast differentiation ability was measured by counting tartrate-resistant acid phosphatase (TRAP) positive multinucleated osteoclasts (defined as those with ≥3 nuclei. *Stat5a* deletion, however, had no effect on osteoclast differentiation. The numbers of TRAP-positive multinucleated osteoclasts showed no difference between *Stat5a*^*-/-*^ and wild-type specimens (Supplementary Figures S5a and b). Next, multinuclear osteoclasts were identified in fractured femoral H&E section^28,29^. At 2 weeks post-fracture, the number of multinuclear osteoclasts were higher in *Stat5a*^*-/-*^ mice than wild-type. Intriguingly, at 4 weeks post-fracture, the multinuclear osteoclasts were lower in *Stat5a*^*-/-*^ mice than wild-type (Supplementary Figures S6a and b). To confirm these observations, we performed immunohistochemistry against dendritic cell-specific transmembrane protein (DC-STAMP). DC-STAMP has been reported as an inducer of osteoclastogenesis and core factor for cell–cell fusion of osteoclast^30,31^. As a result, at 4 weeks post-fracture, the DC-STAMP-positive cells were decreased in *Stat5a*^*-/-*^ mice group (Supplementary Figures S6c). These finding supported that the smaller callus at 4 weeks in *Stat5a*^*-/-*^ mice suggests a rapid callus remodeling.

## Discussion

Emerging evidence suggests that Janus-activated kinase (JAK)-STAT signaling plays an important role in osteoblast differentiation and bone formation^27,32,33^. Tajima et al. have reported that inhibition of STAT1 accelerates osteoblast differentiation and bone fracture healing by regulation of OSX^27^, and Levy et al. reported that inhibition of STAT3 results in the augmentation of BMP-induced osteogenic differentiation^32^. Furthermore, chemical inhibitors of STAT1 and STAT3 have been proposed for the treatment of fractures. Also, as Zhou suggested, the inactivation of stat3 by mutation in osteoblast/osteocyte-specific reduced bone formation^34^. Dieudonne et al. showed that STAT5 inhibitor abrogated in vitro osteogenesis induced by E3 ubiquitin-protein ligase CBL (c-CBL) silencing in hBMSCs^25^. They, however, did not explore the isoform-specific functions of STAT5A and STAT5B during osteoblast differentiation. In the present study, we sought to clarify isoform-specific functions of STAT5A and STAT5B and investigate in vivo role of STAT5 in the bone. Surprisingly, we found that inhibition of STAT5A significantly increased osteoblast differentiation in hBMSCs, whereas inhibition of STAT5B showed no effect on osteoblast differentiation (Fig. 2a, b). Thus, we investigated the relationship between STAT5A and osteogenic transcriptional factors and in vivo role of STAT5A for bone formation and fracture healing in murine models.

One study reported that DLX5 is a central regulator of bone homeostasis through direct regulation of osteogenesis^35^. Previous studies have reported that DLX5 is required for RUNX2 expression under BMP signaling^36–38^. We found that ALP activity and DLX5 expression in *Stat5a*^*-/-*^ mBMSCs were higher than those in wild-type mice during osteogenic differentiation (Fig. 4b–f). STAT5A overexpression in *Stat5a*^*-/-*^ mBMSCs was found to decrease DLX5 expression and impair osteogenic differentiation (Fig. 4). Our results further indicated that STAT5A inhibits DLX5 expression and thereby acts as a negative regulator in osteogenesis.

Interestingly, we found seemingly contradictory protein levels of STAT5A and DLX5 during osteogenesis. Specifically, expression of STAT5A was found to increase throughout the process of osteoblast differentiation (Fig. 1a). This increase in STAT5A seems paradoxical because of its negative effects on osteogenesis. The reason for this pattern and its biological significance have not been previously described. An earlier study found that expression of DLX5 is normally decreased in the late stages of osteogenesis and disappears in fully matured bone^39^. Expression of DLX5 is known to impair the ability of hBMSCs to proceed to the last stages of osteogenesis through suppression of osteocalcin, a marker of terminal osteogenic differentiation^6,40,41^. Consistent with this previous report^39^, our results showed that the expression of DLX5 decreased as osteogenesis progressed (Fig. 1a). Taken together, these findings suggest that the increased expression of STAT5A during osteogenesis may provide a homeostatic mechanism that counterbalances the suppressive effects of excessive DLX5 activity during the late stages of osteogenesis.

In our murine model, the skeletal phenotype of *Stat5a*^*-/-*^ embryos was not found to differ from wild-type controls (Supplementary Figure S2), but postnatal *Stat5a*^*-/-*^ mice were found to have greater bone mass than postnatal wild-type mice. The finding of increased bone mass in *Stat5a*^*-/-*^ mice was maintained with aging. Age-related bone loss was partially protected against in *Stat5a*^*-/-*^ mice, and the bone mineral densities of 40-week-old *Stat5a*^*-/-*^ mice were comparable to those of young wild-type mice (Fig. 3d, e). These findings are consistent with several previous studies that have reported that DLX5 expression is related to postnatal bone developments and regulation of bone homeostasis in adulthood^5,42^. Our results suggest that *Stat5* deletion affects postnatal bone formation via regulation of DLX5.

In addition, *Stat5* deletion increased not only trabecular bone density but also cortical bone formation (Fig. 3i–k). The strength of *Stat5a*^*-/-*^ femurs was found to be greater than that of wild-type mice during biomechanical testing (Fig. 3l). We hypothesize that increased DLX5 expression resulting from *Stat5* deletion may especially target periosteal bone modeling and lead to the observed increase in mechanical strength in *Stat5a*^*-/-*^ mouse bones compared to wild-type specimens. This speculation is supported by our results showing that DLX5 expression predominates in periosteal bone (Fig. 5g), possibly due to the presence of greater numbers of osteoblasts at a specific stage of differentiation^7,43,44^. The balance between bone formation and bone resorption is essential for bone homeostasis. In osteoclast differentiation, a DC-STAMP is a crucial factor in osteoclast fusion. Deletion of STAT5A had no effect on osteoclast differentiation in vitro, but the increase in number of osteoclasts and DC-STAMP positive cells in *Stat5a*^*-/-*^ mice had resulted in rapid callus remodeling (Supplementary Figure 6). Overall, our results demonstrated that STAT5A deletion promotes osteoblast activity and that STAT5A plays an important role in regulating bone formation and bone remodeling.

In conclusion, we have described a novel mechanism of STAT5A in bone homeostasis through regulation of the DLX5. In addition, we demonstrated, for the first time, the STAT5A function in hBMSCs during osteogenesis and confirmed its function in in vivo. Taken all together, our findings contribute to understand the mechanism of STAT5A in controlling bone homeostasis and promoting fracture healing. Furthermore, this work may offer a new therapeutic strategy for preventing age-related osteoporosis.

## Materials and methods

### Cell culture and differentiation conditions

Selection of hBMSCs was performed by selecting them according to the cells’ natural tendency to adhere to the culture dish. Primary hBMSCs were cultured in growth medium, Dulbecco’s modified Eagle’s medium-low glucose (DMEM-LG; Gibco, Carlsbad, CA, USA) with 10% fetal bovine serum (FBS; Gibco), 1% antibiotic–antimycotic solution (Invitrogen, Grand Island, NY, USA), and confluence was achieved within 7 days in 5% CO_2_ at 37 °C. For osteogenesis, cells were cultured in DMEM-LG with 1 μM dexamethasone (Sigma, St. Louis, MO, USA), 10 mM β-glycerophosphate (Sigma), and 50 μM ascorbic acid (Sigma) for 14 days. Mouse bone marrow-derived MSCs (mBMSCs) were isolated from the femurs and tibias of 3–5 mice between 10 and 30 weeks of age. mBMSCs were cultured in growth medium with α-MEM (Gibco) with 10% FBS, 1% antibiotic–antimycotic solution, and 2 mM l-glutamine (Invitrogen, Carlsbad, CA, USA) in 5% CO_2_ at 37 °C^45^. To induce osteogenic differentiation, cells were cultured in growth medium with 10 mM β-glycerophosphate and 50 μM ascorbic acid for 8 days. For osteoclastogenesis, mouse bone marrow monocyte cells (mBMMs) were cultured for 3–4 days in α-MEM containing 15 ng/ml mRANKL (R&D systems, MN, USA) and 40 ng/ml mMCSF (R&D systems)^46^.

### Plasmid constructs

Recombinant pcDNA-mStat5a and pcDNA-mStat5b plasmids were kindly gifted by Dr. Hiroko Yamashita (Nagoya City University, Nagoya, Japan). pcDNA-hSTAT5A, pcDNA-hSTAT5B, and pcDNA-hDLX5 were constructed by amplifying each coding region from cDNA of HeLa cells. All promoter vectors were obtained with polymerase chain reaction (PCR) of human genomic DNA. Human DLX5 and STAT5A promoter regions spanning −2208/+142 base pairs (bp) and −2121/+88 bp were inserted into a pGL3 basic vector (Addgene, Cambridge, MA, USA) and named pGL-DLX5 Full and pGL-STAT5A, respectively. pGL-DLX5 Del1–pGL-DLX5 Del4, containing 5′ serial deletions fragments of the DLX5 promoter were cloned by amplifying the regions from −2003 to +142, −1751 to +142, −1254 to +142, and −645 to +142 bp, respectively.

### Gene transfection and luciferase reporter assays

Recombinant plasmids were transfected into hBMSCs using Lipofectamine LTX and Plus Reagent (Invitrogen, Carlsbad, CA, USA) according to the manufacturer’s protocol. The medium was then replaced with 2 ml DMEM-LG with 10% FBS and 1% antibiotic–antimycotic solution or osteogenic medium. After 24 h, the cells were lysed with 200 µl of 1 × passive lysis buffer (Promega, Madison, WI, USA) per tube, and cell debris was removed. Luciferase activity was measured using the Dual-Luciferase Reporter Assay Kit (Promega, Madison, WI, USA). Relative luciferase activity was normalized by renilla activities to adjust for transfection efficiency.

### Inhibition of STAT5A and STAT5B

Synthetic siRNAs for STAT5A, STAT5B, and DLX5 mRNA were purchased from Bioneer (Bioneer, Daejeon, South Korea). 80% confluence hBMSCs were transfected with each siRNA using Lipofectamine LTX (Invitrogen). The cells were then collected and prepared for analysis. The silencing effects of siSTAT5A and siSTAT5B were checked by measuring reduced protein expression of STAT5A and STAT5B. Negative control duplexes (Bioneer, Korea) were used as negative controls. A STAT5 inhibitor which inhibits phosphorylation of STAT5 via binding to the SH2 domain of STAT5 was purchased from Santa Cruz Biotechnology (sc-355979, Dallas, TX, USA).

### Isolation of mRNA and real-time (RT)-PCR analysis

Total RNA was isolated using the RNeasy Mini Kit (Qiagen, Venlo, Netherlands) according to the manufacturer’s protocol. For quantitative RT-PCR, complementary DNA (cDNA) was synthesized by oligo dT_18_ primer (Invitrogen) using the isolated RNA as a template. The Omniscript Reverse-Transcription Kit (Qiagen) was used to synthesize the cDNA, according to the manufacturer’s instructions. RT-PCR was then performed using 2× qPCRBIO SyGreen Mix Hi-Rox (PCR Biosystems, London, UK) and analyzed using the Applied Biosystems (ABI) StepOne Plus System (ABI, Carlsbad, CA, USA). All primers were purchased from Bioneer. The relative expression of each gene was normalized by GAPDH expression. The specific primer pairs are shown in Supplementary Table S1. All reactions were performed in triplicate.

### Western blot analysis and antibodies

Collected cells were washed twice with 1× phosphate buffered saline (PBS) and lysed by the addition of 100 µl of whole cell lysis buffer (60 mM Tris–HCl pH 6.8, 1% SDS) for 10 min at 100 °C. Collected supernatants, following centrifugation at 13,000 rotations/min (rpm) for 10 min, were quantified using a BCA Protein Assay Kit (Thermo Scientific, Rockford, IL, USA). For western blot analysis, 25 µg of protein were separated on 10% SDS–PAGE under reducing conditions. Subsequently, proteins were transferred onto polyvinylidene difluoride (PVDF) membranes (Amersham Pharmacia, Piscataway, NJ, USA) in transfer buffer (1.4% glycine, 20% methanol and 25 mM Tris–HCl, pH 8.3 for 90 min at 70 V). After that, membranes were incubated in blocking solution (1× TBST (50 mM Tris–HCl, 150 mM NaCl, and 0.1% Tween-20) containing 5% skimmed milk powder) for 1 h at room temperature. Then blocked membranes were incubated in 1% skimmed milk powder solution including primary antibodies overnight at 4 °C. Membranes were then washed three times with 1× TBST, followed by incubation with secondary antibodies for 1 h at room temperature. Primary antibodies used for blotting were anti-STAT5 (1:1000, Abcam; ab68465), anti-STAT5A (1:1000, Abcam; ab32043), anti-STAT5B (1:1000, Abcam; ab178941), anti-DLX5 (1:500, Abcam; ab109737), and anti-RUNX2 (1:1000, Millipore; 05–1478). All secondary antibody complexes were visualized by autoradiography using ECL Plus and ECL Western Blotting Detection Systems (Amersham BioSciences, Buckinghamshire, UK). Signal quantification was performed by scanning the autoradiographs. Signal intensities were determined by densitometry analysis using Image J software.

### Chromatin immunoprecipitation (ChIP) analysis

ChIP analysis was performed using a ChIP Assay Kit (Upstate Biotechnology, Charlottesville, VA) according to the manufacturer’s instructions. Briefly, hBMSCs were induced to form osteoblasts for 3 days and the differentiated cells were then cross-linked by the addition of 1% formaldehyde solution for 10 min at room temperature. The cross-linked cells were lysed with SDS lysis buffer and sonicated. DNA–protein complexes were bound using antibodies against STAT5A (1:1000, Abcam; ab32043) or an unspecific rabbit IgG (1:1000, Santa Cruze; sc2027), and these immune complexes were collected by binding with protein A-agarose after an overnight incubation at 4 °C. After the complexes were washed with 1× PBS, DNA was extracted using phenol/chloroform alcohol precipitation and used as a template for amplification of the DNA fragments. The specific primer pairs for the DLX5 promoter are shown in Supplemental Table 2.

### ALP staining and activity

Differentiated cells were fixed in a mixture of citrate working solution (Sigma Aldrich, 854c) and acetone (2:3) for 30 s and washed in distilled water for 1 min. ALP staining was performed using a Fast Violet B Salt Kit (Sigma Aldrich, 815). Cells were placed in ALP staining solution for 30 min and then washed two times with distilled water. ALP activity was measured using a colorimetric assay at day 0. For this assay, cells were washed two times with ice-cold PBS and harvested in 0.5 ml 50 mM Tris–HCl, pH 7.6. After sonication and centrifugation for 15 min at 12,000 rpm, ALP activity of the supernatant was measured by the quantity of *p*-nitrophenylphosphate released/min/µg total protein using a microplate reader (VersaMax™ Microplate Leader, CA, USA) at 405 nm. Total cellular protein amount was determined using the BCA Assay Kit.

### Von kossa staining and Alizarin Red S staining

Differentiated cells were washed twice with PBS and fixed in a mixture of acetone and methanol (1:1) or 70% ethanol for Von Kossa staining or Alizarin Red S staining, respectively. After fixation, cells were washed two times with distilled water and then stained with either 3% silver nitrate solution or 2% Alizarin Red S solution for 30 min, respectively. For quantification of Alizarin Red S staining, the stained samples were distained by 10% (weight/volume) cetylpyridinium chloride for 30 min at room temperature, and then the supernatants were measured using a microplate reader (VersaMax™ Microplate Leader) at 595 nm.

### Mice

*Stat5a* general knockout mice^10^ were gifted from Dr. Lothar Hennighausen (National Institutes of Health, MD, USA). Deletion of the *Stat5a* gene was confirmed by PCR around the substituted neomycin gene site using the specific primer. The study population of *Stat5a*^*-/-*^ mice was generated by intercrossing *Stat5a* heterozygous knockout (*Stat5a*^+/-^) mice. 10, 20, 30, and 40 weeks old male mice were used in this study.

### μCT analysis

For μCT imaging, soft tissue was dissected from mouse femurs and the bones were fixed in 70% ethanol for 24 h at room temperature. The fixed femurs were then analyzed using high-resolution μCT (Skyscan-1076, Skyscan, Kontich, Belgium). Image reconstruction and analysis was then performed using reconstruction software NRecon (version 1.6.6.0, Skyscan) and CT-analyzer software CTAn (version 1.13.2.1, Skyscan), respectively. Measured parameters for epiphyseal trabecular bone were analyzed using three-dimensional (3D) model visualization software CT-Vol (version 2.0, Skyscan). The acquisition setting conditions were followed by an X-ray source voltage of 70 kVp and a current 140 μA. Beam hardening reduction depended on a 0.5 mm-thick aluminum filter. The pixel size was 18 μm: exposure time was 14.7 s; the rotation step was 0.5°; and full rotation occurred over 360°. The quantitative analysis for the trabecular parameters and cortical parameter were measured at the femoral metaphysis (a total of 300 slices) and femoral diaphysis (a total of 100 slices), respectively. The measured 3D bone parameters of 10-week-old mice included the following: total tissue volume (TV), which included volume measurement of both trabecular and cortical bone; trabecular bone volume per tissue volume (BV/TV); trabecular number (Tb. N); trabecular thickness (Tb. Th); trabecular separation (Tb. Sp); BMD; cortical bone volume (bone volume); cortical cross-sectional thickness (Cs. Th); and cortical cross-sectional area (Cs. area).

### Murine fracture model

A standardized mid-diaphyseal fracture was induced in 24 wild-type mice and 24 *Stat5a*^*-/-*^ mice at 6 weeks of age. Mice were anesthetized with Zoletil^®^ (30 mg/kg body weight, Virbac, Carros, France) and Rompun^®^ (10 mg/kg body weight, Bayer Ontario, Canada), administered by intraperitoneal injection. The right hind leg was shaved and disinfected. The femur was then exposed through lateral incision, and severed in the mid-diaphysis using a scalpel. The fractured femur was then fixed using 22-gauge needle. After fixation the surgical wound was irrigated with normal saline and closed by nylon suture. Immediately after fracture creation, post-surgical condition of the femur was measured using µCT. Mice were then sacrificed at 2 and 4 weeks post-procedure (*n* = 16 and *n* = 32 per time point, respectively). There were no complications or animal deaths during surgery or postoperative management. At 2 and 4 weeks post-procedure (*n* = 8 from the *Stat5a*^*-/-*^ group and *n* = 8 from the wild-type group per time point) were used for BMD measurements and µCT analysis of callus formation and mineralization. The other subjects (*n* = 8 in each group) were used for mechanical testing at 4 weeks post-procedure. Calcified callus volume was determined by subtracting cortical bone volume from the total volume. Before mechanical analysis and imaging, femurs were dissected out from the soft tissue and intramedullary pins were removed. The fracture model protocol was approved by the Committee on the Ethics of Animal Experiments.

### Histology and immunohistochemistry

The fractured femurs were removed from eight mice in each group and fixed in 10% formalin solution for 5–7 days at room temperature. Femurs were decalcified in Calci-Clear Rapid^TM^ (National Diagnostics Inc., GA, USA) solution for 3 days at room temperature. Decalcified femurs were then embedded in paraffin blocks. The paraffin sections were dehydrated by passage through an ethanol series, cleared twice in xylene, and embedded in paraffin, after which 5 mm sections were cut using a rotary microtome. Decalcified femoral sections were stained with Masson’s Trichrome, Safranin O, and Fast Green. For immunohistochemistry, antigen retrieval was performed using pH 6.0 citrate buffer for the deparaffinized sections. Sections were blocked with 5% normal goat serum, without PBS and without 0.1% Tween 20 for 1 h at room temperature. Sections were then incubated with DLX5 antibody (1:100, EPR4488, Abcam) and anti-DC-STAMP (1:100, Millipore; MABF39) for 1 h at room temperature and with anti-rabbit and anti-mouse secondary antibodies for 1 h at room temperature. Then the DAB Substrate Kit (ab64238, Abcam) was used for detection of DLX5 and DC-STAMP positive cells on tissue sections.

### Statistical analysis

The numbers of mice used for in vivo experiments were based on our pilot experiments and µCT analysis was performed in a blinded fashion. For checking the normal distributions of the groups, we first performed normality testing using the Shapiro–Wilk method. If normality tests were passed, two-tailed, unpaired Student’s *t*-tests were used for the comparisons between two groups. If normality tests were failed, Mann–Whitney tests were used for the comparisons between two groups. For comparisons between three or four groups, we used one-way ANOVA if normality tests were passed, followed by Tukey’s post hoc test for all pairs of groups. If normality tests were failed, Kruskal–Wallis testing was performed and followed with Dunn’s post hoc test. GraphPad PRISM Software (version 5.0) was used for statistical analysis. *P* < 0.05 was considered statistically significant. *P < 0.05, **P *<* 0.01, ***P *<* 0.001. All experiments were performed in biological triplicate.

### Study approval

hBMSCs were obtained from the posterior iliac crest of 10 adult donors (age range: 21–51 years) after receiving approval from the Institutional Review Board. All experiments with mice were performed in an Animal Care Center and in accordance with Association for Assessment and Accreditation of Laboratory Animal Care (AAALAC) International Guidelines. All animal care and use protocols were approved by the Committee on the Ethics of Animal Experiments.

## Electronic supplementary material

SF1

SF2

SF3

SF4

SF5

SF6

SF legends

Stable
