# Supplementary figures and images for: Inhibition of STAT5A promotes osteogenesis by DLX5 regulation
Source: Cell Death Dis. 2018 Nov 14;9(11):1136. doi: 10.1038/s41419-018-1184-7 (PMC6235898; doi:10.1038/s41419-018-1184-7)

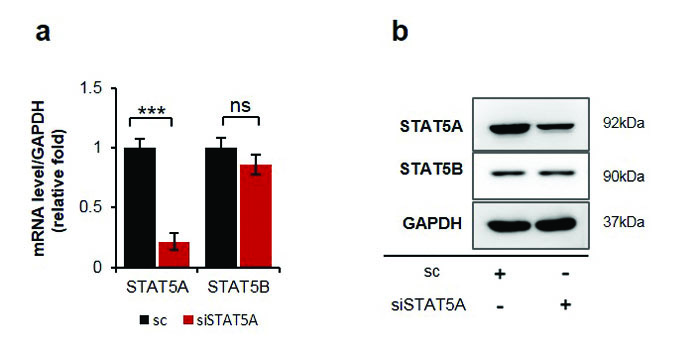

Supplement: Supplementary file 1 — SF1 [file 41419_2018_1184_MOESM1_ESM.jpg]

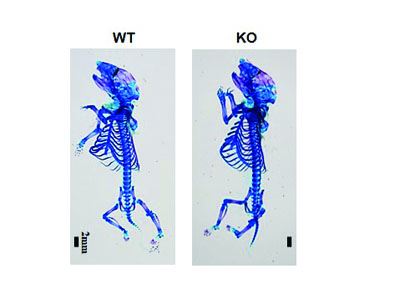

Supplement: Supplementary file 2 — SF2 [file 41419_2018_1184_MOESM2_ESM.jpg]

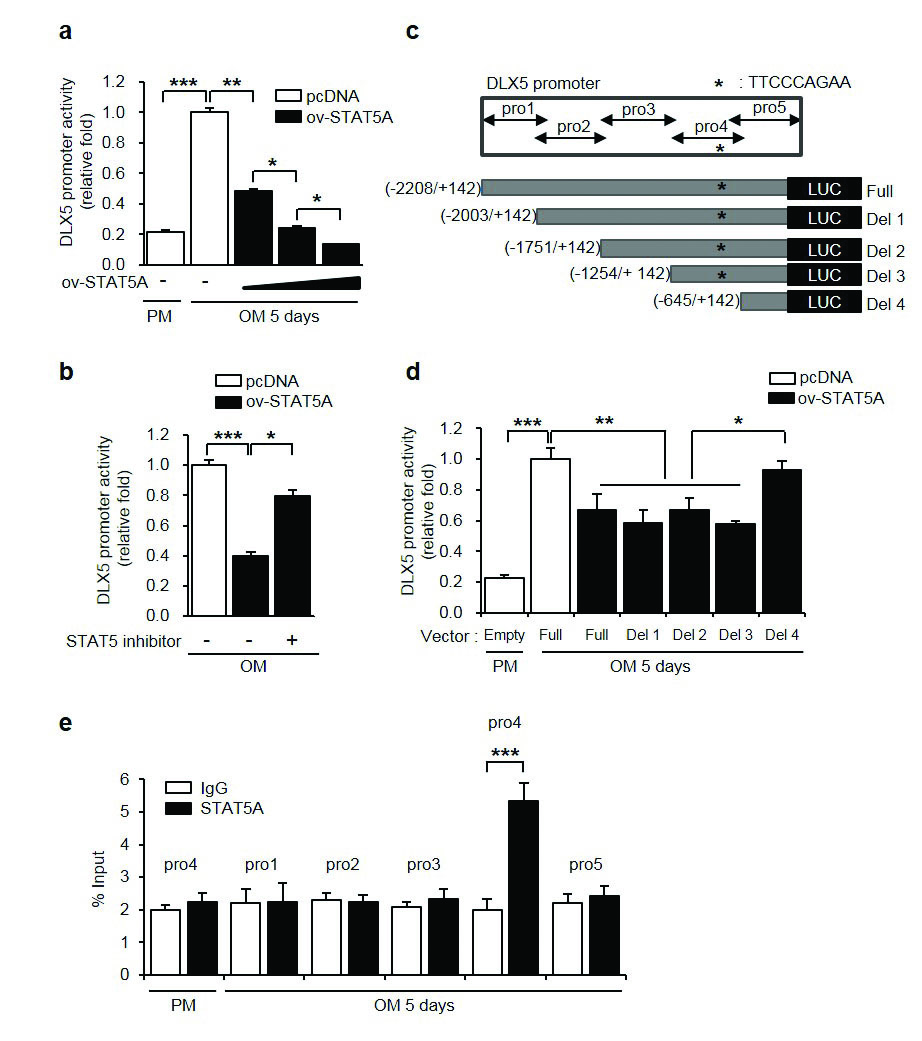

Supplement: Supplementary file 3 — SF3 [file 41419_2018_1184_MOESM3_ESM.jpg]

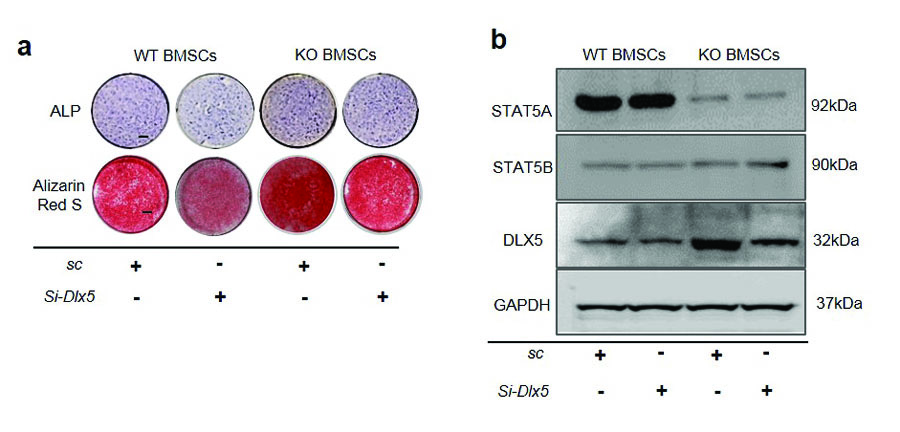

Supplement: Supplementary file 4 — SF4 [file 41419_2018_1184_MOESM4_ESM.jpg]

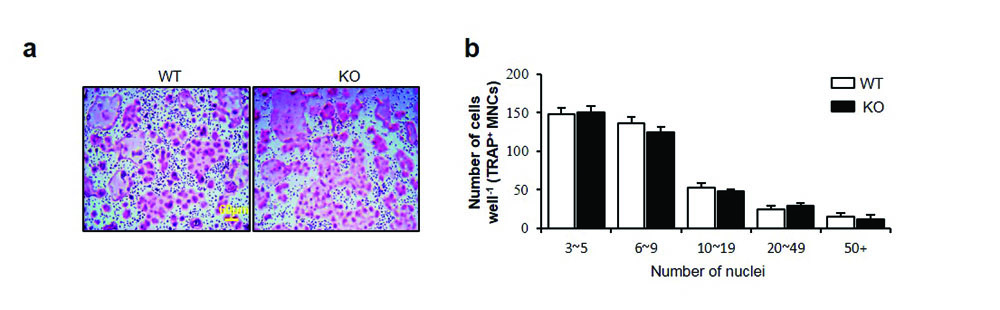

Supplement: Supplementary file 5 — SF5 [file 41419_2018_1184_MOESM5_ESM.jpg]

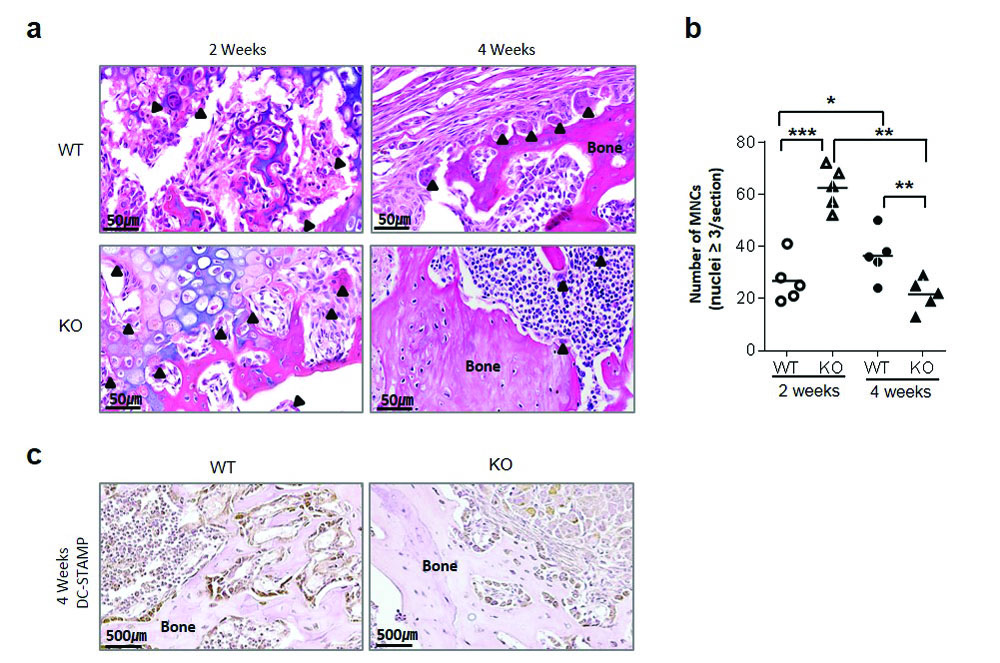

Supplement: Supplementary file 6 — SF6 [file 41419_2018_1184_MOESM6_ESM.jpg]
