# Supplementary material for: Inhibition of STAT5A promotes osteogenesis by DLX5 regulation
Source: Cell Death Dis. 2018 Nov 14;9(11):1136. doi: 10.1038/s41419-018-1184-7 (PMC6235898; doi:10.1038/s41419-018-1184-7)
Supplement: Supplementary file 7 — SF legends [file 41419_2018_1184_MOESM7_ESM.docx]

**Supplementary** **Figure 1.** Effect on the expression of STAT5B by STAT5A suppression using siSTAT5A.(a) Gene expression of STAT5A and STAT5B by siSTAT5A transfection in hBMSCs using real-time PCR. (b) Western blot analysis of protein expression of STAT5A and STAT5B by siSTAT5A transfection in hBMSCs. All mRNA and protein levels were normalized with GAPDH. Each experiment was performed in triplicate (n=3). All error bars indicate ± SEM. ***, P < 0.001.

**Supplementary** **Figure 2.** Skeletal phenotype of wild-type and *Stat5a^-/-^* mice. Whole-mount Alizarin Red S and Alcian Blue staining of embryos (E19.5) and hindlimbs from wild-type (WT) and *Stat5a^-/-^* (KO) mice. Scale bars: 2 mm (*n* = 6 per each group).

**Supplementary Figure 3**. Decreased Transcriptional Activity of DLX5 by STAT5A during Osteogenesis of hBMSCs. **(a)** Relative *DLX5* promoter activity after increasing STAT5A expression. The STAT5A overexpression vector was transfected in quantities ranging from 0 μg to 0.3μg and the total 1ug equalized by adding the remaining amounts of pCDNA3. Luciferase assay was performed on day 4 after plasmid transfection and on day 2 after osteogenesis. PM and OM indicate the growth medium and osteogenic medium respectively. **(b)** Relative *DLX5* promoter activity after STAT5 suppression by STAT5 inhibitor. STAT5 inhibitor was used at concentrations 10 μM. **(c)** A schematic design for the predicted STAT5A binding site on the DLX5 promoter and the DLX5 promoter deletion constructs. The asterisks show the STAT5A binding sequence on the DLX5 promoter region. **(d)** Relative transcriptional activity of truncated DLX5 promoter vectors depending on the presence of the STAT5A binding prediction site. **(e)** The chromatin immune-precipitation assay to assess direct biding site of STAT5A in the DLX5 promoter region. PCR amplification of the combined DLX5 promoter region was performed with specific primers. All experiments were performed in biological triplicate. All data represent mean ±SEM. * *P* < 0.05, ** *P* < 0.01, *** *P* < 0.001.

**Supplementary Figure 4**. Reduced osteoblast differentiation in *Stat5a^-/-^* mBMSCs via down-regulation of DLX5. (a) ALP and Alizarin red S staining of 10-week-old wild-type and *Stat5a^-/-^* mBMSCs upon suppression of DLX5 using *siDlx5* at day 3 and 10 after osteogenic induction, respectively, as indicated. Scale bar, 60 μm. (b) protein levels of STAT5A, STAT5B, and DLX5 depending on suppression reduced *Dlx5* expression in wild-type and *Stat5a^-/-^* mBMSCs. 50μM *siDlx5* was transfected in mBMSCs. Protein levels were checked at day 5 after osteogenesis. Each experiment was performed in triplicate (n=3).

**Supplementary Figure 5.** No difference in osteoclast differentiation *in vitro.* (a) At day 4 after osteoclastogenic induction with M-CSF (40 ng ml^-1^) and mRANKL (15 ng ml^-1^), RANKL-induced osteoclast formation was assayed using 10-week-old male wild-type (WT) and *Stat5a^-/-^* (KO) mBMMs by TRAP staining. Scale bar, 60 μm. (b) Quantification of TRAP-positive multinucleated cells (nuclei ≥ 3) derived from wild-type and *Stat5a^-/-^* mBMMs. All experiments were performed in biological triplicate. All data represent mean ±SEM. Parametric data were analysed using a two-tailed Student’s *t*-test. Nonparametric data were analysed with a Mann–Whitney test.

**Supplementary Figure 6.** Increased osteoclast differentiation in regenerated bone of *Stat5a^-/-^* mice. (a) H&E staining of fractured fumurs from wild-type and *Stat5a^-/-^* mice at 2 and 4 weeks post-fracture. Arrows represent multinucleated cells (nuclei ≥3). (b) Quantification of the number of multinucleated cells (nuclei ≥3)/4mm^2^ of section (n=5 per group). (c) Immunohistochemistry against DC-STAMP at 4 weeks post-fracture of wild-type and *Stat5a^-/-^* mice in the fractured femoral section. All data represent mean ± S.E.M. * *P* < 0.05, ** *P* < 0.01, *** *P* < 0.001.
