# Supplementary material for: Inhibition of STAT5A promotes osteogenesis by DLX5 regulation
Source: Cell Death Dis. 2018 Nov 14;9(11):1136. doi: 10.1038/s41419-018-1184-7 (PMC6235898; doi:10.1038/s41419-018-1184-7)
Supplement: Supplementary file 8 — Stable [file 41419_2018_1184_MOESM8_ESM.docx]

**Supplementary Table 1: A list of primers used for RT-PCR in this study**

| Gene symbol |  | Sequences (5'→ 3') |
| --- | --- | --- |
| STAT5A | Forward | CAGTGGTTTGACGGGGTGAT |
|  | Reverse | GTCGTGGGCCTGTTGCTTAT |
| STAT5B | Forward | ACTGCTAAAGCTGTTGATGGATAC |
|  | Reverse | TGAGTCAGGGTTCTGTGGGTA |
| DLX5 | Forward | GAGTAGGTGTCCCGCCTCAGAACCC |
|  | Reverse | CCAACCAGCCAGAGAAAGAA |
| RUNX2 | Forward | TACAAACCATACCCAGTCCCTGTTT |
|  | Reverse | AGTGCTCTAACCACAGTCCATGCA |
| BSP | Forward | ATACCATCTCACACCAGTTAGAATG |
|  | Reverse | AACAGCGTAAAAGTGTTCCTATTTC |
| GAPDH | Forward | CTGCTGATGCCCCCATGTTC |
|  | Reverse | ACCTTGGCCAGGGGTGCTAA |
| *Stat5a* | Forward | ATGGGGA CTATGATC CAGGC |
|  | Reverse | CCCAGCTTGATCTTCAGCAA |
| *Stat5b* | Forward | GGACTCCGTCCTTGATACCG |
|  | Reverse | TCCATCGTGTCTTCCAGATCG |
| *Dlx5* | Forward | GCTAGATGGGCTACTTTCTCTT |
|  | Reverse | GCGTTCAAACATCCCCGTATGA |
| *Alp* | Forward | CACAATATCAAGGATATCGACGTGA |
|  | Reverse | ACATCAGTTCTGTTCTTCGGGTACA |
| *Bsp* | Forward | CCGGCCACGCTACTTTCTT |
|  | Reverse | TGGACTGGAAACCGTTTCAGA |
| *Opn* | Forward | GCCGAGGTGATAGTGTGGTT |
|  | Reverse | TGAGGTGATGTCCTCGTCTG |
| *Ocn* | Forward | AGCAAAGGTGCAGCCTTTGT |
|  | Reverse | CTTCACTACCTCGCTGCCCT |
| *Gapdh* | Forward | GTGTTCCTACCCCCAATGTGT |
|  | Reverse | ATTGTCATACCAGGAAATGAGCTT |

**Table S2: Specific primer sequences for ChIP analysis**

| Primer name |  | Sequences (5'→ 3') |
| --- | --- | --- |
| Pro 1 | Forward | CTAGCAAGCAGTTTGCAACC |
|  | Reverse | GGCGAATGAAGCATTCACAC |
| Pro 2 | Forward | TACTCCATCGCTCCCAACTG |
|  | Reverse | GGTTGCAAACTGCTTGCTAG |
| Pro 3 | Forward | CCCTCCTTTTGTTTACTTTGG |
|  | Reverse | CAGTTGGGAGCGATGGAGTA |
| Pro 4 | Forward | CATGCAGGAGGATTACCT |
|  | Reverse | CAAATGTCCAGAACCTTTTCAG |
| Pro 5 | Forward | AGCAATGGAGAA GCAAGATACC |
|  | Reverse | GCAGGTAATCCTCCTGCATG |
